# AIRR Community Standardized Representations for Annotated Immune Repertoires

**DOI:** 10.3389/fimmu.2018.02206

**Published:** 2018-09-28

**Authors:** Jason Anthony Vander Heiden, Susanna Marquez, Nishanth Marthandan, Syed Ahmad Chan Bukhari, Christian E. Busse, Brian Corrie, Uri Hershberg, Steven H. Kleinstein, Frederick A. Matsen IV, Duncan K. Ralph, Aaron M. Rosenfeld, Chaim A. Schramm, The AIRR Community, Scott Christley, Uri Laserson

**Affiliations:** ^1^Department of Neurology, Yale School of Medicine, New Haven, CT, United States; ^2^Department of Pathology, Yale School of Medicine, New Haven, CT, United States; ^3^Department of Molecular Biology and Biochemistry, Simon Fraser University, Burnaby, BC, Canada; ^4^Division of B Cell Immunology, German Cancer Research Center (DKFZ), Heidelberg, Germany; ^5^Department of Biological Sciences, Simon Fraser University, Burnaby, BC, Canada; ^6^School of Biomedical Engineering, Science and Health Systems, Drexel University, Philadelphia, PA, United States; ^7^Department of Microbiology and Immunology, College of Medicine, Drexel University, Philadelphia, PA, United States; ^8^Department of Human Biology, Faculty of Sciences, University of Haifa, Haifa, Israel; ^9^Interdepartmental Program in Computational Biology and Bioinformatics, Yale University, New Haven, CT, United States; ^10^Fred Hutchinson Cancer Research Center, Seattle, WA, United States; ^11^Vaccine Research Center, National Institute of Allergy and Infectious Diseases, National Institutes of Health, Bethesda, MD, United States; ^12^Department of Clinical Sciences, UT Southwestern Medical Center, Dallas, TX, United States; ^13^Department of Genetics and Genomic Sciences and Precision Immunology Institute, Icahn School of Medicine at Mount Sinai, New York, NY, United States

**Keywords:** antibody, immunoglobulin, T cell, B cell, immunology, repertoire, AIRR-seq, Rep-Seq

## Abstract

Increased interest in the immune system's involvement in pathophysiological phenomena coupled with decreased DNA sequencing costs have led to an explosion of antibody and T cell receptor sequencing data collectively termed “adaptive immune receptor repertoire sequencing” (AIRR-seq or Rep-Seq). The AIRR Community has been actively working to standardize protocols, metadata, formats, APIs, and other guidelines to promote open and reproducible studies of the immune repertoire. In this paper, we describe the work of the AIRR Community's Data Representation Working Group to develop standardized data representations for storing and sharing annotated antibody and T cell receptor data. Our file format emphasizes ease-of-use, accessibility, scalability to large data sets, and a commitment to open and transparent science. It is composed of a tab-delimited format with a specific schema. Several popular repertoire analysis tools and data repositories already utilize this AIRR-seq data format. We hope that others will follow suit in the interest of promoting interoperable standards.

## Rationale

The increasing use of next-generation sequencing technology to study antibody (IG) and T cell receptor (TR) repertoires led to the establishment of the Adaptive Immune Receptor Repertoire (AIRR) Community in 2015. The goal of the AIRR Community (which was incorporated into The Antibody Society in 2017 to amplify its membership and activities) is to promote community-driven best-practices around the generation, use, and sharing of AIRR sequencing (AIRR-seq or Rep-Seq) data (1). A major goal of the AIRR Community is to facilitate comparative and integrative analyses of AIRR data. So far, the community effort has defined a list of minimal metadata elements (MiAIRR) for describing published AIRR-seq datasets (2) and is actively developing simple interfaces for depositing these datasets in established repositories (3). As a first step toward standardization, the MiAIRR data standard focuses primarily on metadata describing the study design and the type of information to be collected. Providing a standardized machine-readable format, as described herein, will remove a substantial barrier to cross-repository interoperability and cross-dataset analyses. With the proliferation of software tools for the analysis of AIRR-seq data (4–6), there is a pressing need to be able to share data between different applications, pipelines, and databases. To bridge these gaps, the AIRR Community has tasked the Data Representation Working Group (DRWG) to develop data models, schema specifications, file formats, and application programming interfaces (APIs) to promote interoperability and reusability of AIRR-seq data. This paper has two goals: (i) a description of the guiding philosophy we have adopted for defining data representations and (ii) a description of the schema and associated file format we have released specifically for annotated rearrangement data.

## Design goals

Standardized file formats are key to interoperability and effective data sharing of high-throughput AIRR-seq data because they function as a grammar that provides structure to a potentially large set of heterogeneous data. One of the challenges of developing a standard is finding the right balance between rigor and usability that will lead to wide community adoption. The format has to allow the accurate representation of the complexity of the experiment while maintaining flexibility and human-friendliness. The formats and schema developed by the DRWG have been designed to promote accessibility, scalability, and transparency, especially in light of the rapidly changing technological landscape.

### Accessibility

A major goal is to make AIRR-seq data sets the easiest to use for the broadest possible set of researchers and applications. Our primary specification is a relational-compatible schema for commonly used objects in AIRR-seq, which are stored as tab-delimited text files. There exist an enormous number of tools for processing such tabular data supporting a range of expertise levels and applications. Non-programmers can use common spreadsheet applications like Microsoft Excel or Google Sheets to perform simple exploratory data analysis. Programmers can process datasets and perform more complex analyses using flexible and fully-featured environments like R and Python. Large production operations can make data available through SQL databases or through the cloud using distributed computing frameworks like Hadoop and Apache Spark. The key idea is that all of these tools trivially support the ingestion and processing of tab-delimited text data. The tradeoff in this design choice is that we are restricted to a less expressive tabular data model, in contrast to formats like XML, JSON, or Protocol Buffers. Text data also requires parsing different data types, in contrast to binary formats like Apache Parquet. A further goal is compliance with the tidy data structure philosophy (7) wherein all columns are variables and each row contains a single observation of those variables. A tidy structure simplifies analyses employing split-apply-combine strategies and is readily importable into tabular databases. An additional benefit to a tabular format is that it is readily extensible by simply appending columns when a tool or database requires custom fields.

### Scalability

The continued increase in DNA sequencing throughput, combined with increasing interest in the immune repertoire, anticipates the generation of massive AIRR-seq datasets. Indeed, multiple projects propose the generation of billions of IG/TR sequences over the next several years with the intent to mine them for biomarkers, vaccine design, and many other applications. While most analyses of AIRR-seq data today are typically performed in single-node environments by loading data into memory (e.g., via R's data.frame or Python's pandas.DataFrame), the scale of future datasets will likely require the use of distributed computing. A key design consideration in choosing a line-oriented format is therefore to ensure our data files are splittable. Splittable data formats are such that a process can start reading a file from any arbitrary byte position in the file and find the correct record boundaries. This allows a system to read a single, large file from multiple start points in parallel, rather than requiring a process to read data from the beginning of a file. Similarly, it is simple to consider a collection of tab-delimited files with a compatible schema as a single dataset by logically concatenating them, allowing the parallelized writing of datasets.

Importantly, certain compression schemes (e.g., gzip) are not splittable, while others do allow reading from arbitrary byte offsets (e.g., bzip2, blocked gzip). We strongly encourage the use of splittable compression formats. One way in which our accessibility and usability goals might conflict with scalability is our preference for tidy data structures, which necessarily introduces redundancy and may require reshaping of data as a preprocessing step to certain computations. On the other hand, redundancy compresses well. We leave open the possibility of endorsing the use of a binary container format for tabular data, including columnar schemes like Apache Parquet (https://parquet.apache.org/) in the future. Finally, our group is coordinating with the AIRR Community's Common Repository Working Group (CRWG) to define a compatible API for repositories containing large volumes of AIRR-seq data.

### Transparency

The DRWG develops implementations openly on GitHub and we welcome the participation of the community. We are using software engineering best-practices, including continuous integration and delivery to ensure our standards, libraries, and documentation remain consistent. Our format is continuing to evolve and we do not wish to require users to repeatedly reformat possibly large sets of data. Therefore, we have implemented a variation of the semantic versioning scheme (https://semver.org) to ensure that no changes to field definitions occur without a corresponding change in the version number (X.Y.Z). Specifically, because the development repository contains the work of multiple AIRR Community working groups, the major version number (X) is reserved for changes that impact multiple standards, such as updates to the MiAIRR data standard; the minor version number (Y) reflects changes in the schemas and APIs; and the patch version number (Z) is for updates to the associated software packages or documentation that are not accompanied by schema modifications. To further maintain backward-compatibility, a key design goal is that the definitions and names of fields will not be changed unless a major flaw has been revealed. Rather, the schema changes will be preferentially introduced by adding fields with new names and deprecating obsolete fields.

**Figure 1:**
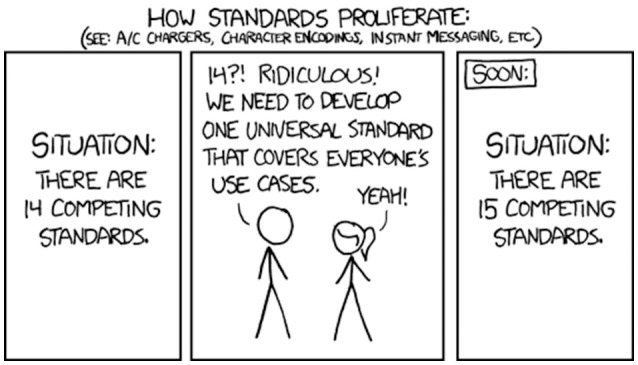
Standards proliferation. The DRWG has been actively engaging as many community members as possible to drive adoption of our new standard. https://xkcd.com/927/.

Adoption is critical to the success of any format. Bioinformatics is plagued with format conversion, and we are wary of simply defining yet-another-format for AIRR-seq data without a clear path to adoption (Figure 1).

To that end, we have developed reference APIs for both R and Python to facilitate addition of the format to existing tools (see section AIRR reference APIs for further details). Furthermore, we have engaged a broad community of authors of popular AIRR software packages and resources to contribute in the design and implementation of the annotated rearrangement schema described herein, including IgBLAST (8), Immcantation (9, 10), iReceptor (11), VDJServer (12), SONAR (13), ImmuneDB (14, 15), TRIgS (16), Partis (17), MiXCR (18, 19), IGoR (20), OLGA (21), and Vidjil (22, 23) (Table 1). Direct involvement of the stakeholders will help ensure our standards continue to evolve to meet the needs of the community. We will continue active outreach to new tool and database developers as part of the AIRR Community's broader efforts.

**Table 1:** Tools and databases supporting the AIRR Rearrangement schema.

**Software**	**Version**	**Support**
AIRR Python Library	1.2	Input, output and validation
AIRR R Library	1.2	Input, output and validation
IgBLAST	1.10	Output
IGoR	TBD	Input and output
Immcantation:Change-O	0.4.2	Input, output and conversion
ImmuneDB	0.24.0	Output
iReceptor	2.0	Input, output and conversion
MiXCR	2.2.1	Output
OLGA	TBD	Input and output
Partis	TBD	Output
SONAR	3.0	Output
TRIgS	2	Input
VDJServer	1.2.0	Input and output
Vidjil-algo	2018.10	Output
Vidjil Web Platform	TBD	Input and conversion

## Analogous efforts

There exist a multitude of standardization efforts in bioinformatics. Indeed, FAIRsharing (24) is a centralized registry of standards, databases, and policies containing over 500 standards related to the life sciences alone (including MiAIRR). In this section, we review some analogous efforts and cover some existing formats that we believe are not suitable for our goals.

### Minimal reporting standards

There exist a large array of “minimal standards” in different life sciences domains that strive to capture necessary information for other research groups to fully reproduce each other's experiments and analyze each other's data (25). For example, the MIAME (Minimum Information About a Microarray Experiment) standard (26) describes the six components of information necessary to describe a microarray experiment, including the study design, the array design, the experimental conditions of hybridization, a description of the biomaterial sample, the actual raw data, and any normalizations. Analogously, the MINSEQE (Minimum Information about a high-throughput SEQuencing Experiment) standard (27) enumerates the five elements of experimental description which are necessary to interpret a high-throughput nucleotide sequencing experiment.

Reporting information about AIRR-seq experiments is unique because datasets may represent samples of B cells and T cells from a variety of different cell types. Furthermore, other standards do not take into account the unique genetic architecture of the IG and TR loci. To address these issues, the AIRR Community has defined its own set of minimal standards [MiAIRR; see (2)]. Most importantly, like many of the other minimal standards efforts, the MiAIRR data standard defines what should be reported, but not how it should be reported, and certainly not in a machine-readable format. In an effort to follow the FAIR principles for data management and promote interoperability, we describe herein our efforts at a machine-readable file format for AIRR-seq experiments that is compliant with MiAIRR.

### Bioinformatics file formats

Here we review a number of commonly used bioinformatics file formats, including which design features we emulated and which design elements are not appropriate for storing AIRR-seq data.

At its core, annotation of IG and TR sequences is derived from alignments against a reference database or an analogous operation. The SAM and BAM formats are ubiquitous for storing aligned NGS data [(28) and https://samtools.github.io/hts-specs/]. However, the genetic architecture of IG and TR sequences requires that each read be separately aligned to the reference set of individual V, D, and J genes. This would require multiple SAM/BAM records per IG/TR sequence, complicating data processing. Furthermore, a given BAM file is mandated to be globally sorted relative to a reference set of contigs, effectively partitioning all V, D, and J alignments into separate parts of the file (or into separate files entirely). The BAM format also implements a custom binary format which requires maintenance of a large toolchain in order to manipulate. Its non-canonical structure has led to considerable effort in porting its toolchain to achieve compatibility with Hadoop-based architectures (29).

Similarly to the VCF format for storing genome variation, we chose an easily readable tab-delimited text-based format. However, VCF files are actually structured into three sections. The meta-information section contains information about the version of the VCF and optional lines about processing of the data. The header section contains the standardized field names for the data captured within each column of the third section, along with additional lines specifying how to parse certain columns. The data section captures the genomic variations per sequence at each line. However, because VCF includes certain fields that have a user-defined structure, these fields must be parsed, leading to considerable complications in interpreting such files. Finally, VCF files tend to grow horizontally (i.e., more samples requires more columns), which is a barrier to scalable architectures that generally assume only the ability to append data.

Another set of common bioinformatics formats are designed to store range annotations on genomes, including BED (30), GFF, and GTF (31). They are also text-based delimited formats. However, their column-set is highly constrained so that a single record contains only a single annotation. To store AIRR-seq data, each IG or TR would have to span multiple lines, complicating the processing of such files and sacrificing a degree of human readability. Furthermore, a significant number of IG/TR annotations are not keyed to genomic coordinates. Finally, these architectures would necessitate storing the sequences themselves in separate files and do not have a natural way to store alignments.

### Other general-purpose container file formats

Accessibility is one of the primary design goals of our format, which strongly suggests using a standard general-purpose storage format for AIRR-seq data. Both JSON and XML are standard formats with parsers in every language that support the description of complicated data records, including nested data. However, both JSON and XML are very verbose (as field names must be replicated into each record), and XML in particular is notoriously finicky to parse, in addition to being unsplittable. Moreover, enforcing the use of a particular schema would be more difficult. Most significantly, necessitating the use of JSON/XML would exclude less computationally-savvy users that depend on spreadsheet software, and preclude the use of many popular statistical tools that assume a tabular data model.

Another family of general-purpose container formats are built around the serialization frameworks in the Hadoop ecosystem, such as Protocol Buffers, Thrift, Avro, and Parquet (32). These are binary file formats that support the use of either tabular or nested data models. The tools can strictly enforce a particular schema and can achieve very high performance, including from the use of columnar storage (33). However, they are not as user-friendly because they require special tools for reading/writing the data and do not have ubiquitous language support.

SQLite represents another option for tabular data storage with broad language support, including the ability to run SQL queries. However, similar to the binary formats above, this would eliminate ease-of-use and require users to use the SQLite API.

### IG- and TR-specific formats

Our work was heavily influenced by previous attempts at developing formats for IG and TR sequences, including VDJML, the output of IMGT/HighV-QUEST (34), and the Change-O format. Indeed, our working group includes members of several of these previous efforts. For the reasons described below, it was decided a new annotated rearrangement format was required to meet the needs of the broader community.

VDJML is an XML-based file format specifically designed for AIRR-seq data and describes the alignments of rearranged sequences to germline genes with the accompanying set of annotations (35). It only represents annotations directly related to the alignment and does not represent the additional downstream annotations. We considered enhancing VDJML to include those annotations, as the expressivity of XML allows a large number of annotations to be stored in a nested structure for each record. However, based on the downsides of XML described above, we ultimately decided that VDJML was not a suitable format. We provide a mapping between the VDJML tags and the data elements in the AIRR Rearrangement schema in Supplementary Table S1.

IMGT provides a text-based serialization format designed for storing annotated IG and TR data that is a variation on the INSDC format (like GenBank and EMBL formats). However, this format is difficult to parse and incompatible with many standard tools for analyzing data. The IMGT/HighV-QUEST tool for annotating IG and TR sequences also provides output in a tabular delimited format. However, the results are spread across multiple TSV files that must be manually joined, including duplicate field names with content that differs between files, which complicates analyses. IMGT's format is also not openly developed, breaking our requirement for transparency.

The Change-O delimited format was most similar to our ultimate design, as it has an IG/TR-specific schema and meets many of our design goals. However, similar to IMGT's tabular format, the Change-O format was designed to meet the needs of a specific tool suite (Immcantation), and therefore lacks some requirements germane to support for a broad range of software tools. Ultimately, due to MiAIRR compatibility requirements, the need for features to support the efforts of other AIRR working groups (e.g., CRWG APIs), and backwards-incompatible technical choices (e.g., end vs. length fields, CIGAR vs. BTOP), we decided to specify a new schema under the AIRR umbrella. In large part, our schema represents a superset of the data elements defined by the Change-O format, with the exception of a few elements that were excluded due to their inapplicability outside Immcantation. A complete correspondence of the fields between the AIRR Rearrangement schema, the Change-O format, VDJML, and IMGT/HighV-QUEST's tabular output is shown in Supplementary Table S1.

## AIRR data representation for annotated rearrangements

We propose a versioned data representation standard for reference alignments and rearrangement annotations for AIRR-seq data using a tab-separated values (TSV) format with a well-defined schema of column names, data types, and encodings for reference alignment results and common upstream/downstream non-alignment annotations. This paper describes v1.2.0 of the data representation standard. The schema is provided in a machine-readable YAML document that follows the OpenAPI v2.0 specification. Strict typing enables interoperability and data sharing between different AIRR-seq analysis tools and repositories, and we are considering the use of controlled vocabularies for certain fields as well. We define a dataset in this context as: a TSV file, a TSV with a companion YAML file containing metadata, or a directory containing multiple TSV files and YAML files. The v1.2.0 schema, TSV format specification, and an example data file are provided in the Supplementary Materials (Supplemental Data Sheet 1).

### AIRR rearrangement schema specification

**Figure 2:**
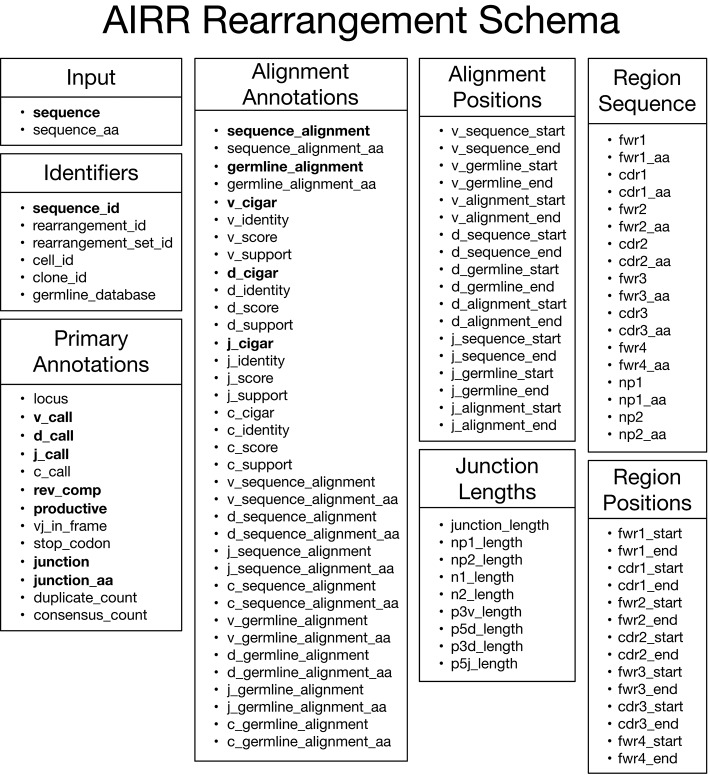
AIRR Rearrangement schema v1.2.0. Overview of the schema for representing annotated rearrangements. Fields in bold are required columns in the TSV. All fields, including those that are required columns in the TSV header, can be set to null by assigning an empty string as the value.

The main data type of interest is an “annotated rearrangement,” which describes a rearranged adaptive immune receptor chain (e.g., antibody heavy chain or TCR beta chain) along with a host of annotations. These data elements are defined by the AIRR Rearrangement schema, which comprises eight categories as shown in Figure 2. By default, data elements representing sequences in the schema contain nucleotide sequences except for data elements ending in “_aa,” which are amino acid translations of the associated nucleotide sequence. The *Input* category consists of the input sequence to the V(D)J assignment process. The *Primary Annotations* category consists of the primary outputs of the V(D)J assignment process, which includes the gene locus, V, D, J, and C gene calls, various flags, V(D)J junction sequence, copy number (duplicate count), and the number of reads contributing to a consensus input sequence (consensus count). The *Alignment Annotations* and *Alignment Positions* categories contain detailed alignment annotations including the input and germline sequences used in the alignment; score, identity, statistical support (E-value, likelihood, etc); the alignment itself through CIGAR strings for each aligned gene; and start/end positions for genes in both the input and germline sequences. The *Region Sequence* and *Region Positions* categories consists of sequence and positional annotations for the framework regions (FWRs) and complementarity-determining regions (CDRs). Lastly, the *Junction Lengths* category provides lengths for junction sub-regions associated with aspects of the V(D)J recombination process. The online documentation (https://docs.airr-community.org) will always have the most in-depth and up-to-date description of the format.

The specification includes two classes of fields. Those that are required and those that are optional. Required is defined as a column that must be present in the header of the TSV. Optional is defined as column that may, or may not, appear in the TSV. All fields, including required fields, are nullable by assigning an empty string as the value. There are no requirements for column ordering in the schema, although the Python and R reference APIs enforce ordering for the sake of generating predictable output. The set of optional fields that provide alignment and region coordinates (“_start” and “_end” fields) are defined as 1-based closed intervals, similar to the SAM, VCF, GFF, IMGT, and INDSC formats (GenBank, ENA, and DDJB; http://www.insdc.org).

Most fields have strict definitions for the values that they contain. However, some commonly provided information cannot be standardized across diverse toolchains, so a small selection of fields have context-dependent definitions. In particular, these context-dependent fields include the optional “_score,” “_identity,” and “_support” fields used for assessing the quality of alignments which vary considerably in definition based on the methodology used. Similarly, the “_alignment” fields require strict alignment between the corresponding observed and germline sequences, but the manner in which that alignment is conveyed is somewhat flexible in that it allows for any numbering scheme (e.g., IMGT or KABAT) or lack thereof.

While the format contains an extensive list of reserved field names, there are no restrictions on inclusion of custom fields in the TSV file, provided such custom fields have a unique name. Furthermore, suggestions for extending the format with additional reserved names are welcomed through the issue tracker on the GitHub repository (https://github.com/airr-community/airr-standards).

### AIRR reference APIs

One of our key design principles was simple programmatic access to the data using commonly-available parsers for tab-delimited formats. While the AIRR Rearrangement schema is fully functional and portable using this approach, we have also implemented Python and R reference libraries that perform type conversion and validate standards compliance for applications that require strict adherence. These libraries also provide a programmatic interface to the entire MiAIRR annotation set and the experimental schemas that are currently under development. These APIs, with bundled schema definitions, are available for download from the AIRR Standards GitHub repository (https://github.com/airr-community/airr-standards), the Comprehensive R Archive Network (https://cran.r-project.org/web/packages/airr), and the Python Package Index (https://pypi.org/project/airr) under a permissive license (CC BY 4.0).

Furthermore, the specification of the AIRR Rearrangement schema using OpenAPI v2.0 provides a standards based mechanism for describing the interface to tools and resources that share AIRR-seq data through APIs. For example, it is possible to utilize automatic documentation and code generation tools such as those found on https://swagger.io to develop web-based AIRR-seq client and server applications.

### AIRR rearrangement schema implementations and support

Several AIRR-seq analysis tools and data repositories have already implemented the AIRR Rearrangement schema while several others are planning support for a future release (see Table 1 for a complete list). An updated list of software and resources that support the various AIRR standards is maintained on the documentation site (https://docs.airr-community.org).

### Example use case

**Figure 3:**
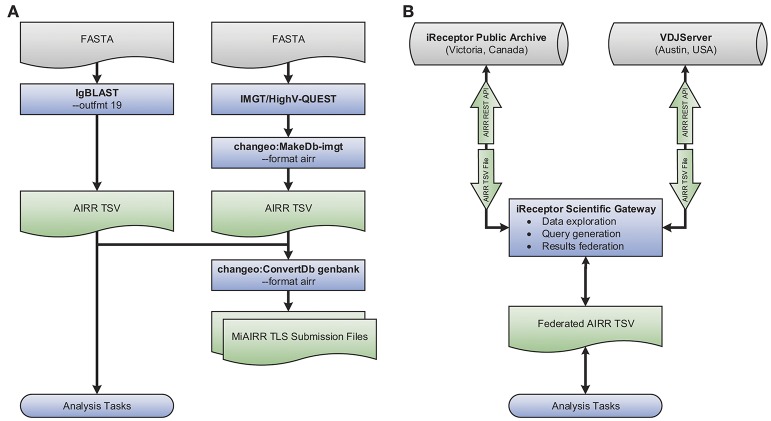
Interoperability example. Shown is a set of flowcharts depicting examples of the interoperability facilitated by the AIRR Rearrangement schema. **(A)** Starting with repertoire sequencing data in the FASTA format, either IgBLAST or IMGT/HighV-QUEST in combination with Change-O's conversion tool may be used. Once data conforms to the AIRR Rearrangement schema, Change-O can be used to generated MiAIRR-compliant GenBank/TLS submissions. AIRR-seq data from separate tools and pipelines can easily be combined for aggregate analysis. **(B)** Data may be exported from or imported to the iReceptor or VDJServer repositories using the TSV format. Data is returned from queries to the separate repositories using the TSV format and can be integrated into a single collection for downstream analysis.

An example use case showcasing the tool interoperability provided by the AIRR Rearrangement schema is shown in Figure 3A. The flowchart demonstrates generating annotated AIRR-seq data with IgBLAST along with additional data processed by IMGT/HighV-QUEST and converting the combined data into an AIRR Rearrangement compatible TSV using Change-O (part of the Immcantation framework). Finally, the merged output of these two distinct tools is used to (a) perform analysis and (b) create MiAIRR-compliant GenBank/TLS submission files. More details regarding each step, the commands used, and an example data set are available from the documentation site (https://docs.airr-community.org).

A further example of the power of the AIRR Rearrangement schema is the ability to perform federated queries across repositories that adhere to the REST API being developed by the CRWG (section Roadmap). For example, the iReceptor Scientific Gateway can search for data of interest (e.g., twin and non-twin sibling data) from multiple studies and across multiple repositories (e.g., the VDJServer and iReceptor Public Archive repositories). Because both repositories support the AIRR Rearrangement schema and provide their output in the TSV format, the gateway can collate those results and further process them into a format suitable for downstream analysis. Such a use case is shown pictorially in Figure 3B and is described in detail in (11).

## Discussion

In collaboration with many stakeholders, we have defined a schema and associated file format for representing annotated IG/TR rearrangements. By choosing to use a ubiquitous tabular container format (TSV), we have ensured that data coming from AIRR-seq pipelines will be available in a way that is accessible to a broad population and will scale to massive data sizes. We have developed this machine-readable format in coordination with other AIRR working groups on GitHub with the goal of enabling tool and database interoperability guided by the goals of accessibility, scalability, and transparency. We have also laid the groundwork for defining additional schemas for AIRR-seq related objects in the future.

The DRWG is engaged in continuous dialog and coordination of efforts with other AIRR Community working groups. We have coordinatedd with the Minimal Standards Working Group to use the MiAIRR data standard as a guide for classifying certain fields as required or optional. We are coordinating with the CRWG to ensure our schema is compatible with the REST API they are developing. The DRWG is also working with the Germline Database Working Group to ensure compatibility with their strategies for curating newly discovered germline reference genes and alleles derived from allele inference tools and sequencing projects. As the AIRR Community effort develops, further data representations will be released to meet these needs. A partial list of schemas under active development and scheduled for near-term release are described in the Roadmap sections that follow.

### Roadmap: detailed alignment schema

A core intermediate step in annotating AIRR-seq data is generating possible alignments of the IG/TR sequences to standard germline databases. While many researchers may be primarily interested in only the optimal reference alignment annotations described by the AIRR Rearrangement schema, some applications also require a list of sub-optimal reference alignments. As such, we are developing an additional TSV specification specifically for representing multiple annotation assignments on a single query sequence as a hit table, similar to the output of tools such as BLAST. Typically, this type of data set will be used as intermediate output, for tasks such as performance evaluation of an alignment tool, reassignment of optimal gene calls using alternative criteria, or performing genotyping with ambiguous gene assignments as a starting guide (36–38). This Alignment schema is available on the main AIRR standards documentation site (https://docs.airr-community.org) under the Data Representations / Alignment Schema section. This specification is in an experimental state, but under active development, and we expect to release an official draft late in 2018.

### Roadmap: metadata schema

Along with the primary data files, a dataset may contain metadata corresponding to the MiAIRR description of the experiment. This may include, but is not limited to, study design, sample demographic data, various experimental conditions, analysis tool versions, and pipeline provenance data. Representing both MiAIRR defined metadata and provenance is somewhat more complex because it contains a hierarchy of relationships that cannot be easily encoded in a tabular format. In this case, we recommend the storage of such data using YAML, a human-friendly superset of JSON. YAML/JSON metadata can be easily modified using a text editor and parsed in virtually every programming language.

The AIRR Metadata schema is also under active development at the time of writing. Currently, a full specification of MiAIRR data elements is complete and available online at the AIRR Standards GitHub repository (https://github.com/airr-community/airr-standards). Completion of the data representation schema and associated API is planned for a future release.

### Roadmap: AIRR data commons

The CRWG has developed a set of recommendations (https://github.com/airr-community/common-repo-wg/blob/master/recommendations.md) for an AIRR Data Commons that promotes the deposition, sharing, and use of AIRR-seq data. The recommendations (i) state the general principles for sharing of AIRR-seq data; (ii) outline the characteristics of compliant repositories for data deposit, storage and access; and (iii) describe a distributed model for compliant repositories for AIRR-seq data, linked by a central registry. The integration between the iReceptor platform and the VDJServer repository (Figure 2B) makes use of the AIRR Rearrangement schema as an early version of a REST API for querying AIRR-seq data. CRWG is currently developing a more comprehensive REST API, which will include the AIRR Rearrangement and Metadata schemas. AIRR compliant data repositories will implement a set of recommendations, including a REST API service, thus providing a standardized query capability and interoperable data format for all data repositories part of the AIRR Data Commons. Specifications and reference service implementations will be released through the AIRR standards GitHub repository (https://github.com/airr-community/airr-standards) at a future date.

## Conclusions

We have described the design goals of the AIRR Community's DRWG along with a schema and file format for annotated IG/TR AIRR-seq data. The data representations described herein can function as a standardized communication tool across different parts of the AIRR-seq data ecosystem, including users, data repositories, and analysis tools. We hope that our guiding design principles of accessibility, scalability, and transparency will help promote wide adoption. We welcome and actively encourage contributions and involvement from the broader community with the ultimate goal of simplifying tool interoperability and data sharing in the study of adaptive immune receptor repertoires.

## Author contributions

All authors contributed work in researching/designing the described standard. JV and SC led the implementation and writing effort. SC and UL functioned as co-chairs of the working group.

### Conflict of interest statement

The authors declare that the research was conducted in the absence of any commercial or financial relationships that could be construed as a potential conflict of interest.
