# Supplementary material for: AIRR Community Standardized Representations for Annotated Immune Repertoires
Source: Front Immunol. 2018 Sep 28;9:2206. doi: 10.3389/fimmu.2018.02206 (PMC6173121; doi:10.3389/fimmu.2018.02206)
Supplement: Supplementary file 2 [file Data_Sheet_1.zip › tsv_format_specification_v1.2.pdf]

TSV Format Specification¶

Data for `Rearrangement` or `Alignment` objects are stored as rows in a *tab-delimited* file and should be compatible with any TSV reader. A dataset is defined in this context as: a TSV file, a TSV with a companion YAML file containing metadata, or a directory containing multiple TSV files and YAML files.

Encoding

- The file should be encoded as ASCII or UTF-8.
- Everything is case-sensitive.

Dialect

- The record separator is a newline `\n` and the field separator is a tab `\t`.
- Fields or data should not be quoted.
- A header line with the AIRR-specified column names is always required.
- Values must not contain tab or newline characters.
- Values should avoid `@`, `#`, and quote (`"` or `'`) characters, as the result may be implementation dependent.
- Nested delimiters are not supported by the schema explicitly and should be avoided. However, if multiple values must be reported in a single column for an application specific reason, then the use of a comma as the delimiter is recommended.

File names
AIRR formatted TSV files should end with `.tsv`.

Structure

The data file has two sections in this order:

1. Header. A single line with column names.
2. Data values. One record per line.

A comment section preceding the header (e.g., `#` or `@` blocks) is not part of the specification, but such a section is reserved for potential inclusion in a future release. As such, a comment section should not be included in the file as it *may* be incompatible with a future specification.

Header

A single line containing the column names and specifying the field order. Any field that corresponds to one of the defined fields should use the specified field name.

Required columns

Some of the fields are defined as `required` and therefore must always be present in the header. Note, however, that all columns allow for null values. Therefore, required columns exist to define a core set of fields that are always present in the table structure, but do not mandate that a value be reported.

Custom columns

There are no restrictions on inclusion of additional custom columns in the Rearrangements file, provided such columns do not use the same name as an existing required or optional field. It is recommended that custom fields follow the same naming scheme as existing fields. Meaning, `snake_case` with narrowing scope when read from left to right. For example, `sequence_id` is the "*identifier* of the *query sequence*".

Consider submitting a pull request for a field name reservation to the airr-standards repository if the field may be broadly useful.

Ordering

There are no requirements that fields or records be sorted or ordered in any specific way. However, the field ordering provided by the schema is a recommended default, with top-to-bottom equating to left-to-right.

Data Values

The possible data types are `string`, `boolean`, `number` (floating point), `integer`, and `null`(empty string).

Boolean values

Boolean values must be encoded as `T` for true and `F` for false.

Null values

All fields may contain null values. This includes columns that are described as `required`. A null value should be encoded as an empty string.

Coordinate numbering

All alignment sequence coordinates use the same scheme as IMGT and INSDC (DDBJ, ENA, GenBank), with the exception that partial coordinate information should not be used in favor of simply assigning the start/end of the alignment. Meaning, coordinates should be provided as 1-based values with closed intervals, without the use of `>` or `<` annotations that denoted a partial region.

CIGAR specification

Alignments details are specified using the CIGAR format as defined in the SAM specifications, with some vocabulary restrictions on the use of clipping, skipping and padding operators. The following table defines the valid operator set.

**Operator**	**Description**
=	An identical non-gap character.
X	A differing non-gap character.
M	A positional match in the alignment. This can be either an identical (=) or differing (x) non-gap character.
D	Deletion in the query (gap in the query).
I	Insertion in the query (gap in the reference).
S	Positions that appear in the query, but not the reference. Used exclusively to denote the start position of the alignment in the query. Should precede any N operators.

N	A space in the alignment. Used exclusively to denote the start position of the alignment in the reference. Should follow any S operators.

Note, the use of either the `=`/`X` or `M` syntax is valid, but should be used consistently. While leading `S` and `N` operators are required, tailing `S` and `N` operators are optional.
