# Supplementary material for: AIRR Community Standardized Representations for Annotated Immune Repertoires
Source: Front Immunol. 2018 Sep 28;9:2206. doi: 10.3389/fimmu.2018.02206 (PMC6173121; doi:10.3389/fimmu.2018.02206)
Supplement: Supplementary file 3 [file Data_Sheet_2.pdf]

AIRR Community Standardized Representations for Annotated Immune Repertoires

Jason A. Vander Heiden[*], Susanna Marquez, Nishanth Marthandan, Syed Ahmad Chan Bukhari, Christian E. Busse, Brian Corrie, Uri Hershberg, Steven H. Kleinstein, Frederick A. Matsen IV, Duncan K. Ralph, Aaron M. Rosenfeld, Chaim A. Schramm, The AIRR Community, Scott Christley[*,†] and Uri Laserson[†]

List of AIRR Community members endorsing this manuscript

Name	Affiliation
Abdellali Kelil	Donnelly Centre for Cellular and Biomolecular Research, University of Toronto, Toronto, Canada
Adam Buntzman	Bio5 Institute, University of Arizona, Tucson, USA
Adrian J Shepherd	Biological Sciences, Birkbeck, University of London, London, UK
Alexander Rosenberg	Microbiology, University of Alabama at Birmingham, Birmingham, USA
Andrew Collins	School of Biotechnology and Biomolecular Sciences, University of New South Wales, Sydney, Australia
Anne Eugster	Center for Regenerative Therapies, TU Dresden , Dresden, Germany
Barbera D. C. van Schaik	Bioinformatics Laboratory, Clinical Epidemiology, Biostatistics and Bioinformatics, Academic Medical Center, Amsterdam, The Netherlands
Bjoern Peters	Division of Vaccine Discovery, La Jolla Institute for Allergy and Immunology, La Jolla, USA
Brian Corrie	Department of Biological Sciences, Simon Fraser University, Burnaby, Canada
Brian G. Pierce	Department of Cell Biology and Molecular Genetics, University of Maryland Institute for Bioscience and Biotechnology Research, Rockville, USA
Camila Coelho	National Institute of Allergy and Infectious Diseases, National Institutes of Health, Rockville, USA
Cathrine Scheepers	Centre for HIV and STIs and Department of Virology, National Institute for Communicable Diseases and The University of the Witwatersrand, Johannesburg, South Africa
Christopher Dubay	Earle A. Chiles Research Institute, Providence Cancer Center, Portland, USA
Corey T. Watson	Biochemistry and Molecular Genetics, University of Louisville School of Medicine, Louisville, USA
Daisuke Komura	Department of Genomic Pathology, Tokyo Medical and Dental University, Tokyo, Japan
Daniel Douek	Vaccine Research Center, National Institutes of Health, Bethesda, USA
David Krag	Department of Surgery, University of Vermont, Burlington, USA
Davide Bagnara	Department of Experimental Medicine, University of Genoa, Genoa, Italy
Donald Lee	Biotechnology HPC Software Applications Institute, Frederick, USA

Duncan Ralph	Computational Biology Department, Fred Hutchinson Cancer Research Center, Seattle, USA
Eline Luning Prak	Pathology and Laboratory Medicine, Perelman School of Medicine, University of Pennsylvania, Philadelphia, USA
Enkelejda Miho	Institute for Medical and Analytical Technologies, University of Applied Sciences and Arts Northwestern Switzerland, Muttenz, Switzerland
Felix Breden	Department of Biological Sciences, Simon Fraser University, Burnaby, Canada
Florian Rubelt	Department of Microbiology and Immunology and Institute for Immunity, Transplantation and Infection, Stanford University School of Medicine, Stanford, USA
George Blanck	Molecular Medicine, Morsani College of Medicine, University of South Florida, Tampa, USA
Gunilla Karlsson Hedestam	Microbiology, Tumor and Cell Biology, Karolinska Institutet, Stockholm, Sweden
Gur Yaari	Faculty of engineering, Bar-Ilan University, Ramat Gan, Israel
Hiroto Katoh	Department of Genomic Pathology, Tokyo Medical and Dental University, Tokyo, Japan
Ignacio Sanz	Department of Medicine, Emory School of Medicine, Atlanta, USA
Jacob D. Galson	Kymab Ltd, Cambridge, UK
Jacob S. Sherkow	Innovation Center for Law and Technology, New York Law School, New York, USA
Jamie Scott	Department of Molecular Biology and Biochemistry, Simon Fraser University, Burnaby, Canada
Jean-Philippe Bürckert	BISC Global Inc., Boston, USA
Johanna Olweus	Department of Cancer Immunlogy, Institute for Cancer Research, Oslo University Hospital and the University of Oslo, Oslo, Norway
Johannes Trück	Division of Immunology, University Children's Hospital Zurich, Zurich, Switzerland
Lindsay Cowell	Department of Clinical Sciences, UT Southwestern, Dallas, Texas, USA
Ludvig M. Sollid	Department of Immunology, University of Oslo, Oslo, Norway
Mangul Serghei	Institute for Quantitative and Computational Biosciences, University of California, Los Angeles, Los Angeles, USA
Marcelo Brigido	Cellular Biology, University of Brasilia, Brasilia, Brazil
Masahide Yano	Center for Drug Evaluation and Research, US Food and Drug Administration, Silver Spring, USA
Mats Ohlin	Department of Immunotechnology, Lund University, Lund, Sweden
Mikhail Shugay	Center of Life Sciences, Skolkovo Institute of Science and Technology, Moscow, Russia
Nima Nouri	Department of Pathology, Yale School of Medicine, New Haven, USA
Ning Jiang	Biomedical Engineering, University of Texas at Austin, Austin, USA

Peng Qiu	Biomedical Engineering, Georgia Institute of Technology and Emory University, Atlanta, USA
Ramit Mehr	The Mina & Everard Goodman Faculty of Life Sciences, Bar-Ilan University, Ramat-Gan, Israel
Rasmi Thomas	US Military HIV Research Program, Walter Reed Army Institute of Research, Silver Spring, USA
Robert Cook-Deegan	School for the Future of Innovation in Society, Arizona State University, Tempe, USA
Samuel O. Oyola	Animal and Human health Immunogenetics, International Livestock Research Institute, Nairobi, Kenya
Simon DW Frost	Department of Veterinary Medicine, University of Cambridge, Cambridge, UK
Sol Efroni	Faculty of Life Sciences, Bar-Ilan University, Ramat Gan, Israel
Thomas MacCarthy	Applied Math and Statistics, Stony Brook University, Stony Brook, USA
Victor Greiff	Department of Immunology, University of Oslo, Oslo, Norway
William D Lees	Biological Sciences, Birkbeck, University og London, London, UK
William J Faison	Duke Human Vaccine Institute, Duke Univeristy School of Medicine, Durham, USA
Xiao Liu	BGI-Shenzhen, Shenzhen, China
Zhang Shifang	AbHelix, LLC, New Jersey, USA
